# Downregulation of BRCA1-BRCA2-containing complex subunit 3 sensitizes glioma cells to temozolomide

**DOI:** 10.18632/oncotarget.2543

**Published:** 2014-10-29

**Authors:** Kit Man Chai, Chih-Yen Wang, Hung-Jiun Liaw, Kuan-Min Fang, Chung-Shi Yang, Shun-Fen Tzeng

**Affiliations:** ^1^ Department of Life Sciences, College of Bioscience and Biotechnology, National Cheng Kung University, Tainan City, 70101, Taiwan; ^2^ Center for Nanomedicine Research, National Health Research Institutes, Zhunan, 35053, Taiwan

**Keywords:** BRCC3, BRCC36, γH2AX, DNA repair, Glioblastoma

## Abstract

We previously found that BRCA1-BRCA2-containing complex subunit 3 (BRCC3) was highly expressed in tumorigenic rat glioma cells. However, the functional role of BRCC3 in human glioma cells remains to be characterized. This study indicated that the upregulation of BRCC3 expression was induced in two human malignant glioblastoma U251 and A172 cell lines following exposure to the alkylating agent, temozolomide (TMZ). Homologous recombination (HR)-dependent DNA repair-associated genes (i.e. BRCA1, BRCA2, RAD51 and FANCD2) were also increased in U251 and A172 cells after treatment with TMZ. BRCC3 gene knockdown through lentivirus-mediated gene knockdown approach not only significantly reduced the clonogenic and migratory abilities of U251 and A172 cells, but also enhanced their sensitization to TMZ. The increase in phosphorylated H2AX foci (γH2AX) formation, an indicator of DNA damage, persisted in TMZ-treated glioma cells with stable knockdown BRCC3 expression, suggesting that BRCC3 gene deficiency is associated with DNA repair impairment. In summary, we demonstrate that by inducing DNA repair, BRCC3 renders glioma cells resistant to TMZ. The findings point to BRCC3 as a potential target for treatment of alkylating drug-resistant glioma.

## INTRODUCTION

Astrocytoma WHO grade IV, also named glioblastoma multiforme (GBM) is the most common and aggressive form of brain tumors [1–4]. Current treatments for gliomas include radical surgical resection, standard radiation therapy, and/or chemotherapy. Clinical reports have indicated that temozolomide (TMZ) chemotherapy in combination with radiotherapy is able to increase the survival period for GBM patients [5–7]. TMZ therapy alone is also a promising therapeutic for elderly patients with GBMs due to its survival benefit with low toxicity. Therefore, TMZ is used as the current first-line therapeutic agent for gliomas [8, 9]. Nevertheless, the median survival of GBM patients remains less than 2 years, with few surviving more than 5 years [6]. To gain more effective therapeutics, the molecular strategy to promote the action of anti-glioma drugs needs to be developed.

TMZ is an alkylating agent and converted by non-enzymatic reaction in the systemic circulation at the physiological pH to the reactive compound, 3-methyl- (triazen-1-yl)-imidazole-4-carboxamide (MTIC). This agent induces the methylation of the O^6^ position on guanine which is mispaired with thymine to form O^6^MeG-T mismatch in double strand DNA, leading to cell death by blocking DNA replication. It is well-known that TMZ-resistance could be mediated by the action of O^6^-methyl guanine methyltransferase (MGMT) which repairs TMZ-induced DNA lesions via removal of O^6^-methyl group [10]. The components involved in mismatch repair (MMR) such as MLH1 and PMS2 are also involved in TMZ resistance in glioblastoma [11]. Additionally, the three essential molecules for HR-dependent DNA repair pathway in mammalian cells, BRCA1, BRCA2, and RAD51, have been reported to contribute to cellular resistance to alkylating agents [12–15].

BRCC3 (previously known as BRCC36) is one of the deubiquitinating enzymes (DUB) in humans and is encoded in the *BRCC3* gene located at the Xq28 locus. It is classified as a member of the JAMM/MPN^+^ family of zinc metalloproteases that specifically cleaves Lys63-linked polyubiquitin chains [16–19]. BRCC3 is known to serve as a component of the BRCA complex involved in TRF2-dependent telomere protection, which maintains genomic stability under physiological condition [20]. The BRCA complex contains multi-proteins, such as BRCA1, BRCA2, BARD1, RAD51 and RAP80, which regulate diverse processes important for the cellular response to DNA damage [19, 21, 22]. This complex specifically recognizes ‘Lys63′-linked ubiquitinated histone H2A and phosphorylated H2AX (γH2AX) at DNA lesions sites and facilitates the recruitment of other DNA repair proteins to DNA damaged sites for DNA repair [21–23]. The BRCA complex forms and accumulates at DNA damage sites in response to DNA damage induced by radiation and/or alkylating agents [13, 24–26]. The study has demonstrated that BRCC3 depletion prevents the formation of BRCA1 nuclear foci, and subsequently impairs the DNA repair pathway in response to DNA damage by ionizing radiation in breast cancer cells, suggesting that BRCC3 is referred as a potential therapeutic target for breast cancer [27]. Nevertheless, the role of BRCC3 in glioma cells remains elusive.

In this study, we investigated the biological function of BRCC3 in two human malignant glioma (MG) cell lines, U251 and A172 cells that expressed a high level of BRCC3 mRNA and exhibited resistance to TMZ. In addition, treatment with TMZ induced the upregulation of HR-dependent DNA repair genes in U251 and A172 cells, as well as the activation of DNA repair process. To gain insights into the functional role of BRCC3 in glioma cells, we examined glioma cell growth by inhibition of BRCC3 expression in U251 and A172 cells. Our findings provide the important evidence showing that targeting BRCC3 expression can impair DNA repair in U251 and A172 cells and increases sensitization of the glioma cells to the alkylating drugs.

## RESULTS

### BRCC3 expression in human glioma tissues and human glioma cell lines

**Figure 1:**
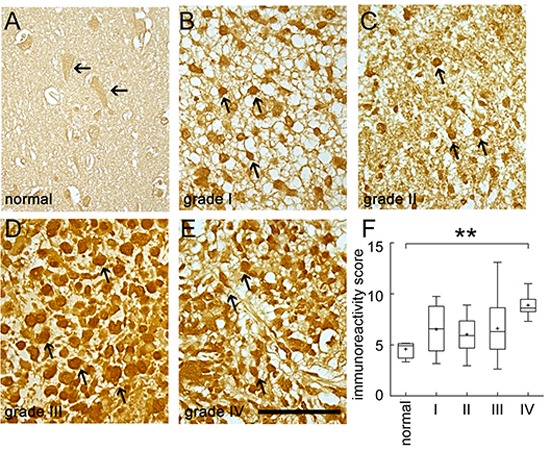
Immunohistochemistry staining for BRCC3 in human brain tumor tissues Human brain tissue slide used for this study contained 24 cases of patients with different grades of gliomas in duplicates. The tissue slide was subjected to immunohistochemistry staining using anti-BRCC3 antibody (Abcam). The representative images show BRCC3 immunoreactivity in normal human cortical tissue **(A)** grade I astrocytoma **(B)** grade II astrocytoma **(C)** grade III anaplastic astrocytoma **(D)** grade IV glioblastoma multiforme **(E)**. Experiments were repeated using anti-BRCC3 antibody from ProSci with similar observations. The staining was photographed under microscope with four images taken from each case. BRCC3 immunoreactivity of normal brain tissue and different grades of glioma were evaluated using ImageJ software **(F)**. Cells with BRCC3 immunostaining were selected through threshold setting of ImageJ software. The data are referred as immunoreactivity score (IRS) representing the average intensity of BRCC3-positive cells normalized over the intensity of background. **p < 0.01, versus normal tissue. Scale bar in A-E, 100 μm.

Through our previous study in genome-wide cDNA expression profiling on tumorigenic C6 glioma cells [28], we found that tumorigenic C6 glioma cells showed abundant amount of BRCC3 (Supporting information Table 1). To determine the functional role of BRCC3 in glioma cells, we first examined the expression of BRCC3 in human glioma tissues. We used the glioma tissue arrays containing tumor sections from human patients with different glioma grades. The results from immunohistochemistry indicated that tumor cells in grade I-III astrocytoma and grade IV GBM displayed a strong BRCC3 immunoreactivity (Fig. 1B-E, arrows), whereas BRCC3 staining was weak in normal brain tissues (Fig. 1A, arrows). Through the analysis of one-way ANOVA, we found that BRCC3 immunoreactivity score (IRS) was significantly correlated to various grades of glioma (*F* = 6.0647, *p* = 0.00295). Moreover, the IRS of BRCC3 in grade IV GBM tissues was higher than normal cortical tissues (Fig. 1F), indicating that the high level of BRCC3 expression is associated with tumor cell growth during glioma progression.

**Figure 2:**
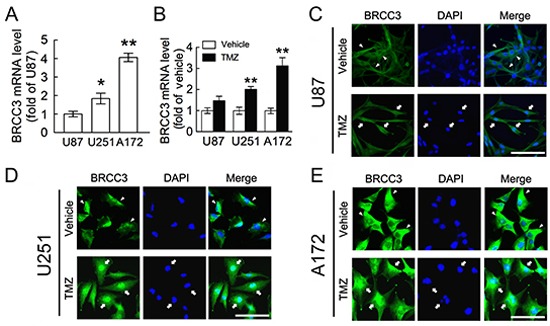
Increase of BRCC3 mRNA expression in resistant glioma cells after TMZ exposure **(A)** Total RNA isolated from U87, U251 and A172 cells was subjected to QPCR analysis for the measurement of BRCC3 mRNA expression. The levels of BRCC3 mRNA expression in U251 and A172 cells were normalized to U87 cells. **(B)** After treatment with TMZ (200 μM) or vehicle for 48 h in DMEM containing 1% FBS, BRCC3 mRNA expression in U87, U251 and A172 cells was analyzed by QPCR. The levels of BRCC3 mRNA expression in the TMZ-treated groups were normalized to their relative control groups (vehicle). (C-E) Immunofluorescence using anti-BRCC3 antibody (Abcam; green) combined with DAPI nuclear counterstaining (blue) was conducted to examine subcellular localization of BRCC3 in U87 **(C)** U251 **(D)** and A172 cells **(E)** at 48 h after TMZ treatment. Experiments were repeated three times with similar observations obtained. Data shown in A and B are means ± SEM of at least three independent experiments. *p < 0.05, **p < 0.01, versus U87 (A); **p < 0.01, versus relative vehicle group (B). Scale bar in C-E, 100 μm.

We then performed *in vitro* study using the three malignant glioblastoma cell lines including U87, U251 and A172 cells. When compared to U87 cells, U251 and A172 cells were more resistant to alkylating agent TMZ (Supporting information Fig. 1A). U251 and A172 cells were also less susceptible to another alkylating agent, BCNU (Supporting information Fig. 1B). Given the fact that BRCC3 is a component of BRCA complex involved in HR-dependent DNA repair for DNA damage [27, 29], BRCC3 mRNA expression in these three glioma cell lines was examined. The results from QPCR analysis showed that U251 and A172 cells expressed higher level of BRCC3 mRNA than U87 cells (Fig. 2A). Exposure to TMZ for 48 h resulted in the upregulation of BRCC3 mRNA expression in U251 and A172 cells (Fig. 2B). Yet, treatment with TMZ caused only a slight upregulation of BRCC3 mRNA expression in U87 cells (Fig. 2B). We also noticed that an increase in BRCC3 mRNA expression was detected in U251 and A172 cells after exposure to BCNU (Supporting information Fig. 1C). Through the examination of the subcellular localization of BRCC3 in U87, U251 and A172 cells, BRCC3 was found in the nucleus of U251 and A172 cells after exposure to TMZ for 48 h (Fig. 2D-E, arrows). BRCC3 was rarely detected in the nucleus of TMZ-treated U87 cells (Fig. 2C, arrows). However, in the absence of TMZ, BRCC3 was predominantly located in the cytoplasm (Fig. 2C-E, arrowheads). The results suggest that BRCC3 is involved in the cellular resistance to alkylating drug-induced cytotoxicity.

### DNA repair-associated gene expression is upregulated in U251 and A172 cells after treatment with TMZ

**Figure 3:**
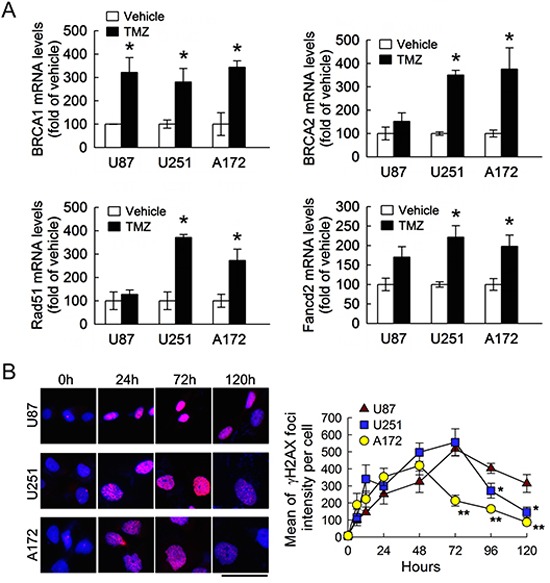
Increase of DNA repair genes and γH2AX foci in glioma cells following TMZ exposure **(A)** Total RNA was isolated from U87, U251 and A172 cells at 48 h after TMZ treatment (200 μM) or vehicle in the medium containing 1% FBS, and then subjected to QPCR analysis for the measurement of DNA repair gene expression indicated as above. Data are means ± SEM of three independent experiments. **(B)** Representative micrographs show γH2AX foci formation in U87, U251 and A172 cells treated by TMZ. The cells were fixed and stained for γH2AX (red) at different time points post exposure to TMZ and DAPI (blue) was used for nucleus counterstaining (left-handed panel). γH2AX foci intensity with indicated time points was quantified by using ImageJ software. Each time point represents the mean of γH2AX foci intensity per cell from five independent fields in each culture (right-handed panel). Experiments were repeated twice and mean ± SEM were shown. *p < 0.05, versus relative vehicle group (A); *p< 0.05, **p< 0.01, versus U87 (B). Scale bar in B, 50 μm.

We were next to examining expression of genes that involved in HR-dependent DNA repair, i.e. BRCA1, BRCA2, RAD51, and FANCD2 [13, 15, 30–32]. As shown in Fig. 3A, the expression of BRCA1, BRCA2, RAD51 and FANCD2 was remarkably increased in U251 and A172 cells at 48 h after treatment with TMZ. Although the expression of BRCA1 in U87 cells was increased in response to TMZ exposure, the change in the expression of the other three DNA repair genes in U87 cells was insignificant. Given the fact that treatment with TMZ promoted the formation of γH2AX foci to provoke the activation of the DNA repair process [33], immunofluorescence was conducted to examine the levels of γH2AX foci in these three cell lines following TMZ. As shown in Fig. 3B, γH2AX foci formation was increased time-dependently in the three cell lines after exposure to TMZ (6, 12, 24 and 48 h). Yet, the intensity of γH2AX foci in A172 cells declined at 72 h after TMZ treatment, whereas the levels of γH2AX foci in U251 cells were also reduced at 96 h post TMZ exposure. The more effective DNA repair occurs in A172 and U251 than U87 cells, suggesting that an increase of DNA repair genes together with BRCC3 in A172 and U251 cells could foster those cells against TMZ-induced cytotoxicity.

### Inhibition of BRCC3 attenuates glioma cell growth

**Figure 4:**
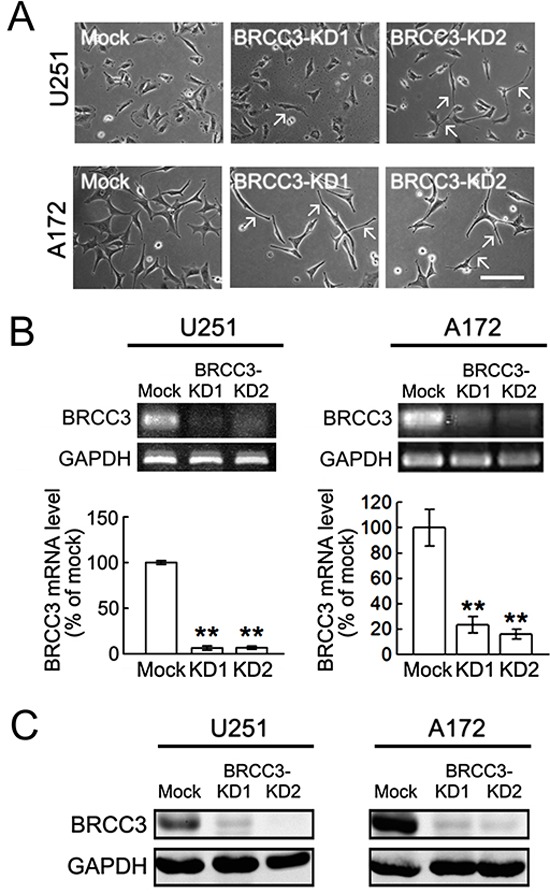
Lentivirus-mediated shRNA targeting BRCC3 in U251 and A172 cells U251 and A172 cells were infected with lenti-ctrl (mock) or lenti-hBRCC3-shRNA_265 (BRCC3-KD1) and lenti-sh-hBRCC3_731 (BRCC3-KD2). The stable infectants were selected in the presence of puromycin as described in Materials and Methods. **(A)** The phase-contrast photographs show the morphological change of U251 and A172 cells with BRCC3-KD1 or BRCC3-KD2 toward bipolar or multipolar enlongated cell shapes (arrows). **(B)** BRCC3 mRNA levels were examined in U251 and A172 cells with BRCC3-KD1 and BRCC3-KD2 using gel-based RT-PCR (upper panel) and QPCR (lower panel). **(C)** Total proteins were isolated from U251 and A172 cells with BRCC3-KD1 and BRCC-KD2, and then subjected to western blot analysis for the measurement of BRCC3 protein levels. The level of GAPDH was used as a loading control. Data shown are means ± SEM of three independent experiments. **p < 0.01, versus mock (B). Scale bar in A, 200 μm.

**Figure 5:**
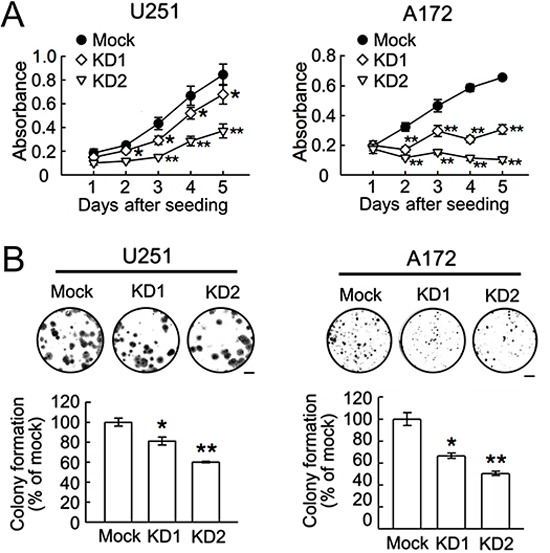
Reduction of cell growth and colony formation ability in U251 and A172 cells by BRCC3 knockdown The stable transfectants (mock, BRCC3-KD1 and BRCC3-KD2) of U251 and A172 cells were seeded at a density of 5×10^3^ cells per well and subjected to MTT proliferation assay **(A)** and colony formation assay **(B)** as described in Materials and Methods. Data are means ± SEM of three independent experiments. *p < 0.05, **p < 0.01, versus mock (A and B). Scale bar in B, 5 mm.

Based on the findings as indicated above that U251 and A172 cells expressed a higher level of BRCC3 than TMZ-sensitive U87 cells, BRCC3 gene knockdown in U251 and A172 cell lines was conducted using lentivirus-mediated shRNA targeting BRCC3 (lenti-sh-hBRCC3). As shown in Fig. 5A, U251 and A172 cells were still viable after infection with lenti-ctrl, lenti-sh-hBRCC3-1 (BRCC3-KD1), or lenti-sh-hBRCC3-2 (BRCC3-KD2). The morphological examination showed that the inhibition of BRCC3 expression induced the elongation of these glioma cell processes (Fig. 4A, arrows). Through QPCR (Fig. 4B) and western blot analysis (Fig. 4C), we found that BRCC3-KD1 and BRCC3-KD2 suppressed the production of BRCC3 mRNA and protein production efficiently in U251 and A172 cells.

**Figure 6:**
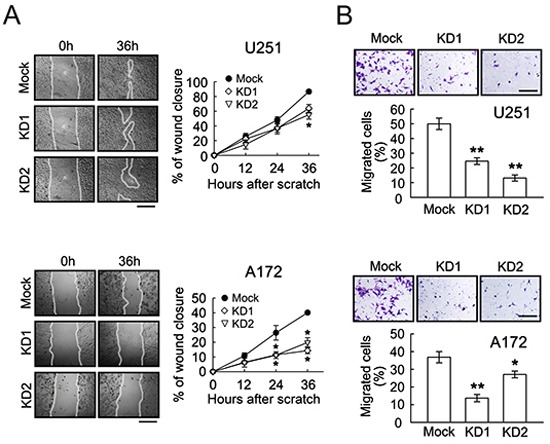
Decrease of cell migration and invasion in U251 and A172 cells by BRCC3 knockdown **(A)** The stable transfectants (mock, BRCC3-KD1 and BRCC3-KD2) of U251 and A172 cells were subjected to cell scratch assay after the cell density of the cultures reached confluence (left-handed panel). The wound closure was quantified at the initial point and the other indicated time points (right-handed panel). The results are represented as the percentage of wound closure by measuring migrating cell covered area over the wound area observed at the initial time point. **(B)** The stable transfectants as indicated above were plated onto the upper side of the transwell insert filters for cell invasion. After 6 h, the cells that migrated to the bottom side of the filter were stained (purple color) and counted. Data shown are means ± SEM of at least three independent experiments (A and B). *p < 0.05, **p < 0.01, versus mock (A and B). Scale bar in A, 500 μm; B, 200 μm.

Further results indicated that the ablation of BRCC3 gene expression effectively reduced the growth rate of U251 and A172 cells (Fig. 5A), and suppressed their clonogenic ability (Fig. 5B). The cell scratch assay indicated that BRCC3 gene knockdown caused a decline in the migration of U251 and A172 cells (Fig. 6A). The cell invasion of the two cell lines was also diminished by the inhibition of BRCC3 expression (Fig.6B). These results further confirm that BRCC3 is essential for the regulation of glioma cell growth, migration, and invasion.

### Downregulation of BRCC3 increases their sensitization to TMZ

**Figure 7:**
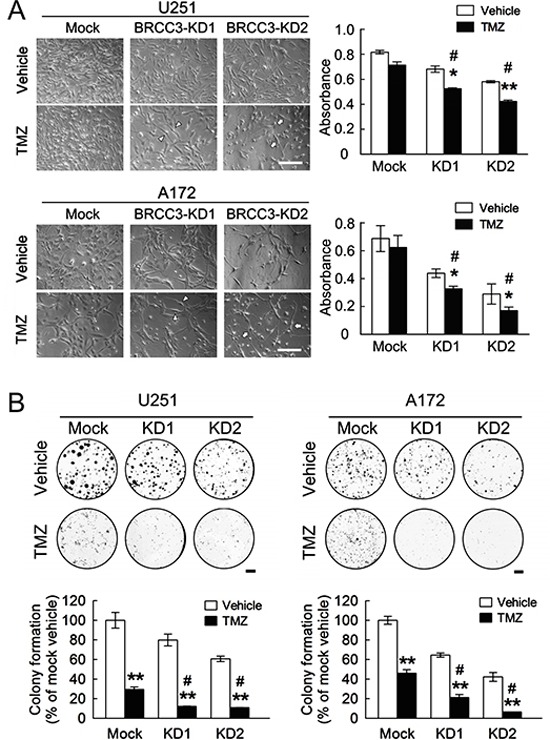
Enhanced sensitization of U251 and A172 cells to TMZ by BRCC3 knockdown **(A)** The stable transfectants (mock, BRCC3-KD1 and BRCC3-KD2) of U251 and A172 cells were seeded at a density of 3×10^4^ per well and treated with 200 μM of TMZ for 48 h in the medium containing 1% FBS, cultures were then subjected to MTT assay for the examination of the cell viability (right-handed panel). The phase-contrast photographs show that U251 and A172 cells with less flat (BRCC3-KD1; arrowheads) and fine bipolar shape (BRCC3-KD2; arrows) were observed at 48 h after TMZ treatment (left-handed panel). **(B)** The stable transfectants as indicated above were seeded onto 6-well culture plates. At day 3 post seeding, cells were then treated with 200 μM of TMZ in the medium containing 10% FBS. The cultures were subjected to colony formation assay in the presence of TMZ for 7 days. Data are means ± SEM of three independent experiments. *p < 0.05, **p < 0.01, versus relative vehicle group (A and B); ^#^*p* < 0.05 versus TMZ-treated mock cells (A and B). Scale bar in A, 200 μm; B, 5 mm.

BRCC3 depletion has been reported to enhance the cytotoxic effect of ionizing radiation on breast cancer cells [27]. Accordingly, we examined if the depletion of BRCC3 promotes TMZ-induced cytotoxicity in U251 and A172 cells. Our data showed that BRCC3 gene knockdown reduced the cell viability of TMZ-treated U251 and A172 cells when compared to the relative TMZ-treated mock cells (Fig. 7A). Moreover, the colony formation of U251 and A172 cells with BRCC3-KD1 and BRCC3-KD2 was decreased in the presence of TMZ compared to TMZ-treated mock (Fig. 7B). Moreover, BRCC3 gene knockdown promoted the cell death of U251 and A172 cells in response to BCNU (Supporting information Fig. 1D). These observations demonstrate that the downregulation of BRCC3 expression increases the sensitization of U251 and A172 cells to alkylating drug-mediated cytotoxicity.

**Figure 8:**
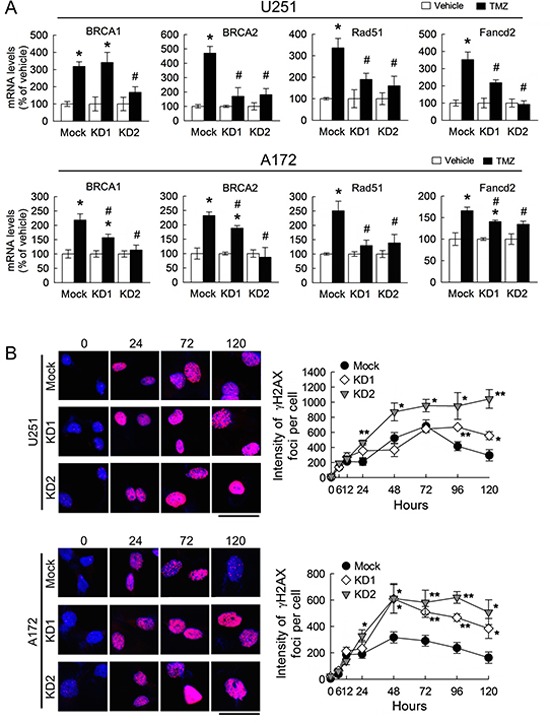
Downregulation of BRCC3 expression alleviates DNA repair ability in U251 and A172 cells Total RNA was isolated from U251 and A172 cells with stable BRCC3 knockdown (BRCC3-KD1 and BRCC3-KD2) at 48 h after treatment with TMZ (200 μM) or vehicle in the medium containing 1% FBS, and then subjected to QPCR analysis for the measurement of the expression of DNA repair genes indicated as above **(A)** Data are means ±SEM of three independent experiments. **(B)** Representative micrographs for γH2AX foci formation induced by TMZ (200 μM) in the medium containing 1% FBS in U251 and A172 cells with BRCC3 inhibition (left-handed panel). The cells were fixed and stained for γH2AX (red) at different time points post TMZ treatment, and DAPI (blue) was used for nucleus counterstaining. Quantification of γH2AX foci intensity with indicated time points (right-handed panel). Each time point represents the mean of γH2AX foci intensity per cell from five independent fields in each culture. Downregulation of BRCC3 disrupts the DNA repair of TMZ-induced toxicity. Experiments were repeated twice and mean ± SEM was shown. *p < 0.05, versus relative vehicle group; ^#^*p* < 0.05, versus mock with TMZ treatment (A), *p < 0.05, **p < 0.01, versus mock (B). Scale bar in B, 50 μm.

In addition, the deficiency of BRCC3 gene expression reduced the levels of DNA repair-associated genes (i.e. BRCA1, BRCA2, RAD51 and FANCD2) in TMZ-treated U251 and A172 cells, when compared to those detected in TMZ-treated mock cells (Fig. 8A). In contrast, the intensity of γH2AX foci in U251 and A172 cells with BRCC3 inhibition remained at higher levels in the presence of TMZ for 48 – 96 h, when compared to TMZ-treated mock cells (Fig. 8B). Moreover, the formation of γH2AX foci expression persisted higher at a later time point of TMZ treatment (120 h) in U251 and A172 cells with BRCC3 gene knockdown than mock cells. The findings reveal that inefficient DNA repair process is resulted from the downregulation of BRCC3 in U251 and A172 cells.

## DISCUSSION

Abnormal expression of BRCC3 has been observed in several breast cancer cell lines and invasive ductal carcinomas [19]. Clinic data have reported that GBM patients with aberrant amplification of BRCC3 gene copies display a trend of low Kaplan-Meier survival probability (National Cancer Institute. 2005. REMBRANDT home page. http://rembrandt.nci.nih.gov. Accessed 2014, February 10). Here, we provide the evidence that the level of BRCC3 expression is much higher in GBM tumor cells compared to normal human brain tissues, and grade I-II astrocytoma. In addition, we show that two glioma cell lines, which derived from patients with grade IV malignant glioma and GBM, U251 and A172 cells, had abundant expression of BRCC3 mRNA.

We show here that U251 and A172 cells are considerably more resistant to TMZ than U87 cells, which is consistent with the findings from others [34, 35]. Similar to the previous observations [35], we notice that three glioma cells exhibited undetectable MGMT mRNA expression in the absence or presence of TMZ (Unpublished observations of Chai et. al.). Yet, U251 and A172 cells display a higher level of BRCC3 expression. Moreover, the expression of BRCC3 in the two glioma cell lines is upregulated in response to TMZ or BCNU. These observations demonstrate that upregulation of BRCC3 expression is the general response of resistant glioma cells to alkylating agents.

Here, we address that BRCC3 exists evidently in the nuclei of U251 and A172 cells after exposure to TMZ, but lesser in U87 cells. Although an increase in the formation of γH2AX foci is induced in the three glioma cell lines after exposure to TMZ, faster decline in the formation of γH2AX foci is observed in U251 and A172 cells rather than U87 cells. This reveals that the activation of DNA repair in U251 and A172 cells is effective. Given the fact that BRCC3 (BRCC36) is essential for the protection of breast cancer cells from ionizing radiation-induced DNA damage [27], a high level of BRCC3 could protect U251 and A172 cells from TMZ-induced cytotoxicity. The proapoptotic effector Bak and antiapoptotic BCL-2 family member Mcl-1 have been reported to participate in TMZ-induced apoptosis of U251 cells [36]. The action of Mcl-1/Bak axis on TMZ-induced cell death of BRCC3-KD glioma cells is not yet known. In addition, it is possible that BRCC3 effect could combine with other factors associated with TMZ resistance in glioblastoma, such as EFEMP1 (Fibulin-3), an extracellular matrix protein [37].

BRCA-triggered DNA repair pathway contains multiple proteins including BRCA1, BRCA2, BARD1, BRCC3 (BRCC36), BRCC45, and RAD51 [19, 27, 38]. Our study presents that BRCA1, BRCA2 and RAD51 are increased in the two glioma cell lines following treatment with TMZ. BRCC3 has been reported to interact with BRCA1 directly to trigger BRCA-associated DNA repair pathway [27]. In addition, BRCA1, BRCA2 and RAD51 are required for HR-dependent DNA repair pathway in TMZ-resistant glioma [12–14]. Thus, an increase in the expression of BRCC3 together with these DNA repair-associated genes following TMZ promotes effective HR-directed DNA repair, which in turn contributes to cellular resistance of U251 and A172 cells to TMZ-induced cytotoxicity. U87 cells, however, may execute insufficient DNA repair mechanism, since only BRCA1 mRNA expression is increased in TMZ-treated U87 cells.

The defect of the FA repair pathway was originally identified in a rare genetic disease, Fanconi Anemia (FA), with several abnormal features including chromosomal instability and hypersensitivity to DNA cross-linking agents such as mitomycin C [39–41]. FANCD2, a critical FA protein, interacts with BRCA1, BRCA2 and RAD51 at the site of DNA damage to facilitate DNA repair [39, 40, 42]. Previous findings have shown that downregulation of FANCD2 expression by siRNA-mediated approach in U87 and T98MG cells can increase cellular sensitivity to glioma chemotherapeutic agents, TMZ and BCNU, demonstrating that FA repair pathway is involved in glioma resistance to DNA alkylating agents [43]. Our results indicate that exposure to TMZ causes a profound expression of FA/BRCA DNA repair genes in U251 and A172 cells when compared to U87 cells. Since U251 and A172 cells express a higher level of BRCC3 than U87 cells either with or without TMZ treatment, it suggests that FA cooperated with BRCA-DNA repair pathways could be highly activated in U251 and A172 cells.

A growing body of evidence indicates that the inhibition of DNA repair gene expression in cancer cells in combination with radiation and/or anti-tumor drugs is a positive therapeutic strategy [19, 25, 29, 44, 45]. For instance, targeting RAD51-dependent repair together with ionizing radiation and TMZ has been reported to be an effective therapeutic strategy for glioma [12]. Interruption of BRCA1 and BRCA2 expression or their function is also found to enhance radiosensitivity and the chemotherapeutic effectiveness of tumor cells [19, 26, 45–47]. Moreover, the knockdown of BRCC3 (BRCC36) in cooperation with ionizing radiation can increase breast tumor cells undergoing apoptosis [27]. Here, to elucidate the functional role of BRCC3 in DNA repair pathway induced by TMZ, we have generated the glioma cell lines with stable BRCC3 gene knockdown. The inhibition of BRCC3 gene expression significantly reduces the cell viability and clonogenicity of U251 and A172 cells by TMZ treatment, indicating that BRCC3 can be treated as the target in order to enhance the sensitivity of glioma cells to TMZ. Moreover, BRCC3 is a direct regulator of BRCA1 activation induced by ionizing radiation [27]. This molecule is required for the recruitment of BRCA1 and other DNA repair proteins to DNA damage sites [44]. The expression level of genes associated with DNA repair in TMZ-treated U251 and A172 cells with BRCC3 gene knockdown is much lower than that detected in TMZ-treated mock cells. In addition, higher levels of γH2AX foci in U251 and A172 cells with inhibition of BRCC3 gene expression persist at a later time point following TMZ exposure when compared to that observed in TMZ-treated mock cells. Thus, the inhibition of BRCC3 expression may impair DNA repair mechanism in U251 and A172 cells, which can promotes sensitization of the two glioma cell lines to TMZ. Given that the formation of γH2AX foci is correlated to cell senescence [48], we have also examined if the inhibition of BRCC3 gene expression could lead to glioma cell senescence. Through immunostaining of senescence associated β-galactosidase (β-Gal), rare β-Gal^+^ cells were observed in mock or BRCC3-KD U251 and A172 cultures (data not shown). We also noticed that the inhibition of BRCC3 sensitizes U251 and A172 cells to BCNU-induced cell toxicity. This supports our view that BRCC3 upregulation is common response to protect glioma cells against alkylating agent-induced DNA damage.

Previous report has demonstrated that abrogation of BRCC3 (BRCC36) using small interfering RNAs targeting BRCC36 causes no significant effect on the cell apoptosis of human breast cancer cell lines, suggesting that the downregulation of BRCC3 expression is not lethal [27]. In contrast, we show here that the lentivirus-mediated knockdown of BRCC3 can induce the morphological change in U251 and A172 cells. Moreover, the depletion of BRCC3 gene expression decreases the cell growth and clonogenic ability of U251 and A172 cells. The cell migration and invasion of U251 and A172 cells are also impaired by BRCC3 knockdown. However, similar levels of γH2AX foci formation are observed in mock and BRCC3-KD glioma cells in the absence of TMZ, indicating no serious DNA damage in BRCC3-KD cells without TMZ. Moreover, we found no significant change in the expression of genes associated DNA repair in mock and BRCC3-KD glioma cells without TMZ treatment (data no shown). These observations reveal that BRCC3 is involved in glioma cell growth even if no alkylating agent-induced DNA damage. Besides of involving in DNA repair, BRCC3 is one of the four-member DUBs to form BRISC (Brcc36-containing isopeptide complex) in the cytosol, which selectively cleaves Lys63-linked polyubiquitin [49, 50]. Recently, BRCC3 in the cytosol has been reported to promote the inflammasome activation by deubiquitinating NLRP3 in LPS-primed macrophages [51]. Notably, BRCC3 is predominantly observed in the cytoplasm of glioma cells without TMZ treatment. Thus, it remains to be further dissected if the DUB activity of cytosolic BRCC3 manipulates glioma cell growth in the absence of alkylating agents.

In summary, BRCC3 is involved in the regulation of cell growth and TMZ resistance in glioma cells. Given the findings that HR-DNA repair proteins (BRCA1, BRCA2 and RAD51) are significantly increased in TMZ-resistant glioma cells, an abundance expression of BRCC3 in glioma cells seems likely to foster HR-dependent DNA repair processes. Thus, effectively inhibiting BRCC3 expression in order to cause an insufficiency of homology-directed repair in glioma cells is a potential therapeutic strategy for sensitizing glioma tumor cells to the alkylating anti-tumor drugs.

## MATERIALS AND METHODS

### Materials

Culture petri dishes were obtained from BD Biosciences. Dulbecco's modified Eagle's medium (DMEM) with high glucose, antibiotics (penicillin/streptomycin), and puromycin were purchased from Invitrogen (Carlsbad, CA, USA). Fetal bovine serum (FBS) was obtained from HyClone Laboratories (Logan, UT, USA). 8.0-μm pore size transwell inserts were purchased from SPL Life Sciences (Korea). Poly- D-lysine (PDL), Temozolomide (TMZ), BCNU (1,3-Bis (2-chloroethyl)-1-nitrosourea,Carmustine), chromogen, 3,3′diaminobenzidine tetrahydrochloride (DAB), 4′,6-diamidino-2-phenylindole (DAPI), DMSO, MTT, proteinase K, and proteinase inhibitors were purchased from Sigma-Aldrich (St. Louis, MO, USA). Vectastain ABC kit, FITC-avidin and Cy3-avidin were purchased from Vector Laboratories (Burlingame, CA, USA). Antibodies used for the study are listed as follow: anti-BRCC3 (Abcam, Cambridge, MA, USA; ProSci Incorporated, Poway, CA, USA) and anti-glyceraldehyde 3-phosphate dehydrogenase (GAPDH; Millipore, Billerica, MA, USA), anti-γH2AX (Millipore), horseradish peroxidase (HRP)-conjugated or biotinylated secondary antibodies (Jackson ImmunoResearch Laboratories, West Grove, PA, USA).

### Cell Culture

Human malignant glioma U87 cell line was obtained from American Type Culture Collection (Rockville, Maryland, USA). U251 and A172 cells were provided from Dr. Pei-Jung Lu (Institute of Clinical Medicine, National Cheng Kung University). The human glioma cell lines were grown in DMEM containing antibiotics and 10% FBS.

### Lentivirus-mediated shRNA targeting BRCC3

The shRNA lentiviral particles against human BRCC3 expression in human MG cell lines were designed by Biosettia Inc (San Diego, CA, USA). The lentivirus vector constructs used in this study included pLV-mU6-[sh-scramble] EF1a-GFP-puromycin (lenti-sh-ctrl), pLV-mU6-[sh-hBRCC3] EF1a-GFP-puromycin (lenti-sh-hBRCC3_265 and lenti-sh-BRCC3_731). Gene transduction and preparation of transfectants were performed as described previously [52]. Briefly, U251 and A172 cells at a density of 3×10^5^ cells per dish were seeded onto 60-mm dishes for 24 h. The cultures were infected by 300 μl of lentiviral particles (lenti-sh-ctrl and lenti- sh-hBRCC3) in 3 ml of DMEM containing 10% FBS, and then incubated for 24 h in 5% of CO_2_ at 37°C. The medium was then replaced by DMEM containing 10% FBS for 24 h. The cultures were subsequently treated using 3 μg/ml puromycin in the presence of 10% FBS in DMEM for 48 h to generate stable transfectants. Cultures transduced with lenti-sh-ctrl were used as the control group (mock). The mock cells and the cells infected by lenti-sh-hBRCC3_265 (BRCC3-KD1) and lenti-sh-BRCC3_731 (BRCC3-KD2) were subjected to QPCR and western blot analysis to determine the efficiency of lentivirus-mediated shRNA against BRCC3 gene expression.

### MTT cell proliferation assay

Cell growth was determined using the MTT colorimetric assay. Cells were seeded at a density of 5×10^3^ - 3×10^4^ per well onto 96- or 24-well plates supplied with DMEM containing 10% FBS. The cultures were treated with 200 μM of TMZ or 100 μM of BCNU in DMEM containing 1% FBS for the distinct time periods. 0.2% DMSO was used as vehicle. After that, MTT solution (5 mg/ml) was added to each well for 4 h, and the purple formazan crystals were dissolved in DMSO solution. Absorbance was measured at 595 nm using an ELISA-plated reader.

### Cell scratch assay

A monolayer scratch assay was performed as previously described [52]. After mechanically scratch with a pipette tip, cells were rinsed with PBS and grown in DMEM without serum for various time periods. The cell motility was analyzed by measurement of wound closure area using ImageJ software (Wayne Rasband, NIMH, Bethesda, MD; http://rsbweb.nih.gov).

### *In vitro* transwell cell invasion assay

24-well format 8.0-μm pore size transwell inserts were used to analyze the cell invasion. Cells were seeded at the density of 2×10^4^ cells per well onto PDL-coated transwell inserts which were placed in the culture wells with DMEM containing 10% FBS in the lower compartment of each well. After 6 h, the inserts were removed from the culture wells and then fixed in 4% paraformaldehyde for 10 min. The inserts were stained with 0.05% crystal violet in ddH_2_O for 10 min and the upper surface of the insert was gently cleansed with cotton swabs. The cells that migrated through the filter membrane toward the other side of the inserts were counted using ImageJ software. The results are represented as the percentage of migrated cells over the total seeded cells per culture.

### Colony formation assay

Colony formation assay was performed by replating cells at a density of 200 cells per well onto a 6-well culture plate in DMEM containing 10% FBS. After 14 days, the cells were fixed with 4% paraformaldehyde for 10 min and then stained by using 0.05% crystal violet in ddH_2_O for 15 min. Alternatively, for examination of clonogenic ability of glioma cells with TMZ treatment, a density of 1000 cells per well were seeded onto a 6-well culture plate and 200 μM of TMZ or vehicle in DMEM containing 10% FBS was added to the cultures at 3 day after seeding. The cultures were continuously maintained for another 7 days and subjected to the colony formation assay. Colonies produced by each cell-group were counted and measured using ImageJ software.

### Quantitative real-time polymerase chain reaction

Quantitative-PCR (QPCR) Light Cycler Fast Start DNA Master SYBR Green used in this study was purchased from Roche Diagnostics (USA). Total RNA isolation, PCR, and data normalization were performed as described previously [52, 53], with minor modifications. PCR reactants were also analyzed on 1% agarose gels to verify primer specificity. The specific primer sequences for the genes were designed using NCBI software Primer-BLAST (http://blast.ncbi.nlm.nih.gov/Blast.cgi). The oligonucleotides were synthesized in MWG Biotech AG (Table 1).

**Table 1:** Primers used for QPCR analysis

Species	Gene	Forward primer (5′ - 3′)	Reverse primer (5′- 3′)
Human	BRCC3	CGTTTGTCTCAACCACGCTC	TTGTATCATCGTTCAACTCCCC
Human	BRCA1	AGCAGAATGGTCAACTGATGAATA	ACTGCTGCTTATAGGTTCAGCTTT
Human	BRCA2	AATTAGCATGTGAGACCATTGAGA	GATTTGTGTAACAAGTTGCAGGAC
Human	FANCD2	ATCTGCTATGATGATGAATGCTGT	AGAGCTGCTTTCTTATCACCAAGT
Human	RAD51	CAGTGATGTCCTGGATAATGTAGC	TTACCACTGCTACACCAAACTCAT
Human	GAPDH	TTGGTATCGTGGAAGGACTCA	TGTCATCATATTTGGCAGGTTT

### Western blot analysis

Total proteins were extracted from the cells using PBS containing 1% NP40, 1% Triton-X 100, 0.1% SDS, 1 mM PMSF and iced proteinase inhibitor cocktail. After centrifugation at 10000 rpm for 10 min, the supernatant was collected and protein concentration was determined using a Bio-Rad DC protein assay kit (Bio-Rad Laboratories, Hercules, CA, USA). 100 μg of the protein in SDS-PAGE loading buffer was heated in boiling water for 5 min, and loaded onto 10% polyacrylamide gel. After electrophoretic transfer to nitrocellulose membranes, the membranes were then immunoblotted with anti-BRCC3 (1:1000) and anti-GAPDH (1:2000) at 4^°^C overnight. An appropriate HRP-conjugated secondary antibody was then added for 1 h at room temperature. The targeted proteins were detected by chemiluminescence using the ECL-Plus detection system (PerkinElmer Life Sciences, Waltham, MA, USA).

### Immunofluorescence

Cells were grown on coverslips at a density of 2×10^4^ cells per well. After treatment with TMZ (200 μM) or vehicle (0.2% DMSO) for distinct time periods in DMEM containing 1% FBS, cells were fixed in 4% paraformaldehyde for 10 min at room temperature and incubated in PBS containing 0.01% Triton X-100 for 20 min. The cultures were reacted with anti-BRCC3 antibody (1:100) or anti-γH2AX (1:500) in PBS containing 5% horse serum at 4°C overnight. The cells were then incubated with biotinylated secondary antibody (1:200) for 1 h at room temperature, followed by FITC-avidin (1:200) or Cy3-avidin (1:800) for 45 min at room temperature. The nuclear counterstaining was performed using DAPI. The immunostained cells were then photographed under a fluorescence microscope (Nikon E800) with a color digital camera.

### Quantification of γH2AX foci

To measure the formation of γH2AX in glioma cells, the images were randomly captured under a 40x objective from five fields per culture with a cooling CCD system. The total γH2AX intensity of each cell was determined using ImageJ software. The results were presented as mean of γH2AX foci per cell by measuring total fluorescence intensity of γH2AX foci over total cells from five independent fields in each culture.

### Immunohistochemistry

Human brain tumor tissue slide (Pantomics Inc., Richmond, CA, USA) was first heated at 60^°^C for 30 min before deparaffinization using xylene. The slide was rehydrated in in various concentrations of ethanol solutions (100%, 95% and 70%) and distilled water. After peroxidase inactivation using 0.03% H_2_O_2_, the slide was incubated with proteinase K (10 μg/ml), followed by incubation of anti-BRCC3 antibody (1:100) at 4^°^C overnight. After the incubation of biotinylated secondary antibody (1:200) for 1 h at room temperature, the immunostaining for BRCC3 was viewed using Vectastain ABC kit (1:200) and DAB. The slide was dehydrated in ethanol solutions (60%, 70%, 95% and 100%) and xylene before mounting.

### Statistical analysis

Data were analyzed for statistical significance by the 2-tailed unpaired Student's t test using Sigma-Plot 10 (SYSTAT Software Inc., San Jose, CA). The results are represented as means ± SEM. The differences between the compared groups are considered statistically significant at *p* < 0.05.

## SUPPLEMENTARY FIGURE AND TABLE


