# Supplementary material for: Downregulation of BRCA1-BRCA2-containing complex subunit 3 sensitizes glioma cells to temozolomide
Source: Oncotarget. 2014 Oct 29;5(21):10901–15. doi: 10.18632/oncotarget.2543 (PMC4279418; doi:10.18632/oncotarget.2543)
Supplement: Supplementary file 1 [file oncotarget-05-10901-s001.pdf]

SUPPLEMENTARY FIGURE AND TABLE

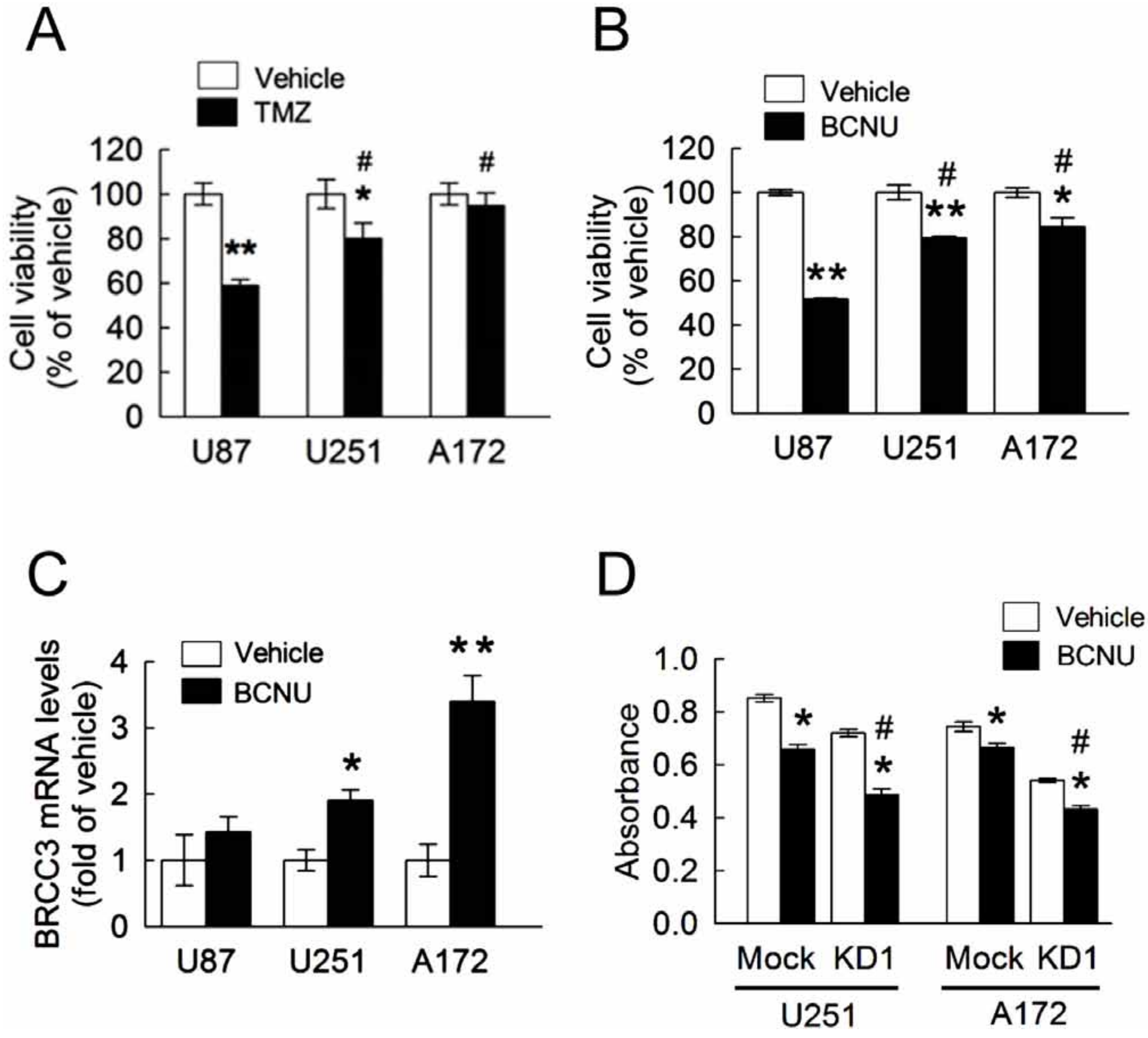

Supplementary Figure S1: Effects of TMZ and BCNU on U251 and A172 cell viability and their BRCC3 expression. **(A, B)** U87, U251 and A172 cells were treated with TMZ (200 µM) or BCNU (100 µM) for 48 h in the medium containing 1% FBS, and then subjected to MTT assay for the examination of the cell viability. **(C)** After treatment with BCNU (100 µM) or DMSO in DMEM containing 1% FBS for 48 h, BRCC3 mRNA expression in U87, U251 and A172 cells were then determined by Q-PCR. The levels of BRCC3 mRNA expression in the BCNU-treated groups were normalized to their relative vehicle group. **(D)** Stable transfectants (mock, BRCC3-KD1 and BRCC3-KD2) of U251 and A172 were subjected to MTT assay after BCNU treatment for 48 h. *$p < 0.05$, **$p < 0.01$, versus vehicle (A-C); $^{\#}p < 0.05$, versus TMZ- or BCNU-treated U87 (A, B); $^{\#}p < 0.05$, versus relative BCNU-treated mock (D).

Supplementary Table S1. List of genes involved in Cellular Response to DNA Damage Stimulus (GO:0006974) with an increased level in tumorigenic C6 cells versus less tumorigenic C6 cells.

Probe Set ID	Gene Symbol	Gene Title	Fold change	*P* value
1374318_at	Brcc3	BRCA1/BRCA2-containing complex, subunit 3	23.54	6.46E-08
1388867_at	MGC112830	similar to transcription factor	2.29	1.03E-06
1368947_at	Gadd45a	growth arrest and DNA-damage-inducible, alpha	2.73	3.02E-06
1388583_at	Cxcl12	chemokine (C-X-C motif) ligand 12 (stromal cell-derived factor 1)	22.40	1.50E-05
1367940_at	Cxcr7	chemokine (C-X-C motif) receptor 7	12.59	2.62E-05
1367664_at	Ankrd1	ankyrin repeat domain 1 (cardiac muscle)	8.60	3.76E-05
1368025_at	Ddit4	DNA-damage-inducible transcript 4	2.43	7.23E-05
1370830_at	Egfr	epidermal growth factor receptor	7.68	7.41E-05
1368341_at	Polb	polymerase (DNA directed), beta	2.26	8.86E-05
1373733_at	Bok	BCL2-related ovarian killer	2.07	9.35E-05
1389735_at	Rps6ka6	ribosomal protein S6 kinase polypeptide 6	24.05	1.08E-04
1382659_at	Pla2r1	phospholipase A2 receptor 1	6.23	1.24E-04
1368308_at	Myc	myelocytomatosis oncogene	2.07	1.77E-04
1368174_at	Egln3	EGL nine homolog 3 (C. elegans)	15.20	2.28E-04
1370912_at	Hspa1b	heat shock 70kD protein 1B (mapped)	2.80	6.22E-04
1370080_at	Hmox1	heme oxygenase (decycling) 1	2.28	1.91E-03
1383439_at	Npas2	neuronal PAS domain protein 2	2.90	2.91E-03
1368311_at	Mgmt	O-6-methylguanine-DNA methyltransferase	2.26	1.51E-02
1369029_at	Plscr1	phospholipid scramblase 1	2.03	4.17E-02
